# Varied diets, including broadleaved forage, are important for a large herbivore species inhabiting highly modified landscapes

**DOI:** 10.1038/s41598-020-58673-5

**Published:** 2020-02-05

**Authors:** Annika M. Felton, Emma Holmström, Jonas Malmsten, Adam Felton, Joris P. G. M. Cromsigt, Lars Edenius, Göran Ericsson, Fredrik Widemo, Hilde K. Wam

**Affiliations:** 1 Southern Swedish Forest Research Centre, Swedish University of Agricultural Sciences, PO Box 49, SE-230 53 Alnarp, Sweden; 2 Department of Wildlife, Fish and Environmental Studies, Swedish University of Agricultural Sciences (SLU), Umeå, SE-901 83 Sweden; 3 Centre for African Conservation Ecology, Department of Zoology, Nelson Mandela University, PO Box 77000, Port Elizabeth, 6031 South Africa; 4 Division of Forestry and Forest Resources, NIBIO, PO Box 115, Ås, NO-1431 Norway

**Keywords:** Biodiversity, Forest ecology

## Abstract

Diet quality is an important determinant of animal survival and reproduction, and can be described as the combination of different food items ingested, and their nutritional composition. For large herbivores, human landscape modifications to vegetation can limit such diet-mixing opportunities. Here we use southern Sweden’s modified landscapes to assess winter diet mixtures (as an indicator of quality) and food availability as drivers of body mass (BM) variation in wild moose (*Alces alces*). We identify plant species found in the rumen of 323 moose harvested in Oct-Feb, and link variation in average calf BM among populations to diets and food availability. Our results show that variation in calf BM correlates with variation in diet composition, diversity, and food availability. A varied diet relatively rich in broadleaves was associated with higher calf BM than a less variable diet dominated by conifers. A diet high in shrubs and sugar/starch rich agricultural crops was associated with intermediate BM. The proportion of young production forest (0–15 yrs) in the landscape, an indicator of food availability, significantly accounted for variation in calf BM. Our findings emphasize the importance of not only diet composition and forage quantity, but also variability in the diets of large free-ranging herbivores.

## Introduction

Eating is complicated. Animals have to trade off a food item’s potential energetic and nutritional gains against the risks of acquisition, such as the increased vulnerability to predation, exposure to plant toxins, or conspecific antagonism^1^. What an individual eats, and where and when it does so, will in turn affect its fitness^2,3^, as diet quality is an important determinant of reproduction and survival in animal populations^4^. For cervids (members of the deer family Cervidae), diet has repeatedly been shown to influence physiological and reproductive fitness^5–7^. The impact of diet on individual fitness can occur through changes in body mass (BM), as well as through maternal nutritional effects^8,9^ that can have flow-on implications for several generations^10^. Diet quality is primarily determined by the combination of different plant items ingested, and each item’s nutritional composition^11^. A high diversity of available food items should enable a balanced intake of nutrients and energy^11^, and the avoidance of high doses of each plant species’ defensive chemicals^12^.

Globally, intensive land management practices are altering an increasing proportion of land area^13,14^. This can cause food resources to become concentrated in space and time^15^, and constrain the ability of cervids to acquire a suitable diet. Even in sparsely inhabited northern Europe, human modification of the landscape has been extensive^16^, with some regions primarily defined by intensive forestry, agriculture, urban environments, and limited protected areas. Humans largely control both the cervids’ food resources and mortality rates. In many regions, this has led to an increase in some cervid populations^17^. Furthermore, in these environments seasonal variation in food abundance can readily compound limits on food resources for cervids. In summary, the cervids of northern Europe need to mix their diets in increasingly modified landscapes, with limited plant diversity, high seasonal variation and potentially inflated competition for the resources that are available.

**Figure 1:**
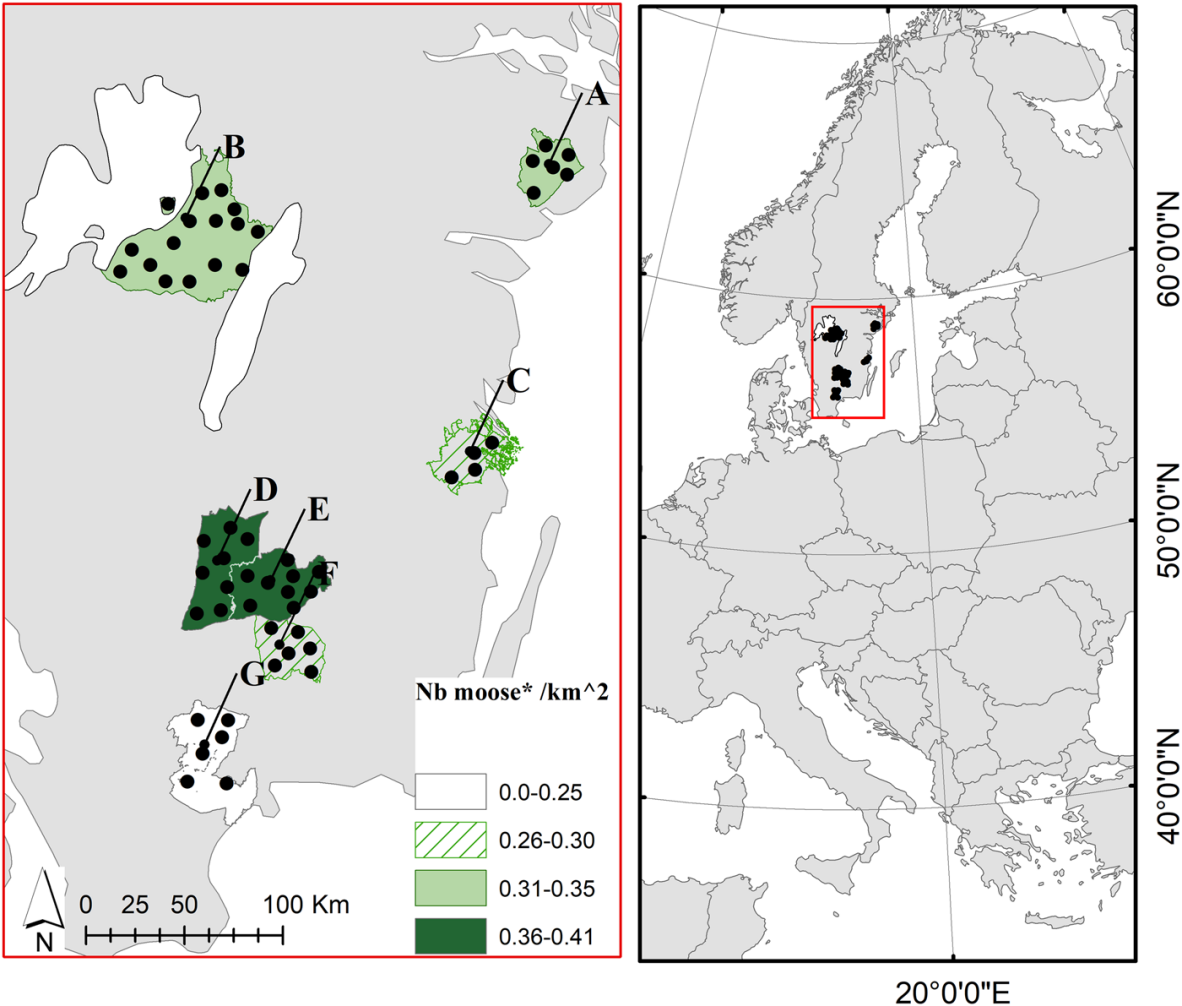
Map of the seven moose management areas (MMA) in Southern Sweden used in this study. Each MMA harbours a moose population and includes several moose management units (MMU, subpopulations), indicated with a black dot. Variation in moose population density is indicated with a coloured scale (*number of moose harvested in the 2014/15 hunt km^−2^; www.viltdata.se). MMA name: A = Södermanland 3; B = V. Götaland 6; C = Västervik S. & Misterhult; D = Jönköping 6; E = Kronoberg 7; F = Kronoberg 4; G = Skåne NÖ.

Here we try to disentangle the implications of diet composition and food availability on body mass in wild populations of moose (*Alces alces*), using multiple areas with varying environmental conditions in the highly modified landscapes of southern Sweden. The moose is an adaptable browser^18^ that uses body stores built up during the growing season to meet the demands of the northern winter^19^. In the spring and summer they selectively consume leaves and forbs with particular nutritional attributes^20^ from many plant species, whereas during winter they eat twigs, needles and bark with lower nutrient content^21^. To assess winter dietary choices across different landscapes, we identified the plant contents found in the rumen of 323 moose from multiple populations and subpopulations across southern Sweden (Fig. 1, Table 1SI). We focused on the winter period (Oct-Feb) as this is when dietary constraints are highest, and when moose hunting allows for sample collection. We link variation in moose calf BM among subpopulations to diets and food availability at the landscape level. We used calf BM as an index of subpopulation nutritional status because (i) conditions in early life have long-lasting effects on demographics^22,23^, often leading to strong correlations between calf BM and calf production in a population^24^ (also tested in this study); (ii) the use of calves circumvents age effects on BM^25,26^; and (iii) calves are less affected by hunter bias in the sex and size of individuals harvested^27^. We hypothesise that more diverse diets should be associated with populations having higher calf BM, and place our findings within the context of the potential benefits of diversifying landscapes in regions homogenized by intensive natural resource management.

## Results

### Diet composition

**Figure 2:**
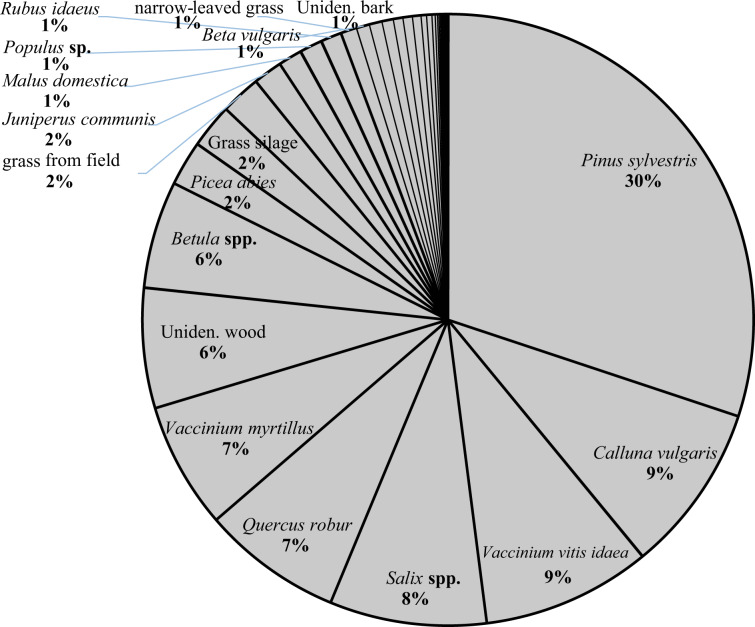
Mean relative abundance of plant categories (44 in total) identified through macroscopy of rumen samples (% of dry matter) from 323 shot moose between 23^rd^ October 2014–22^nd^ of February 2015 in southern Sweden. Plant categories that represent at least 0.7% of total dm are described by name.

In total, we identified 44 different categories of plant food in the rumen samples. The winter (Oct-Feb) diet based on all rumen samples (Fig. 2) was characterised by relatively large proportions (% dry matter (dm)) of twigs from *Pinus sylvestris*, twigs from three dwarf bushes *Vaccinium vitis-idaea*, *Calluna vulgaris* and *V. myrtillus*, and the three broadleaved tree species/genera *Salix* spp, *Quercus robur* and *Betula* spp. In total, 63% of dm was material from trees and bushes, 28% from dwarf shrubs and 9% from forbs, grasses and root vegetables. Four types of supplementary foods were found: sugar- or fodder beets (varieties of *Beta vulgaris*), carrots (*Daucus carota*), potato (*Solanum tuberosum*) and grass silage. We categorize these as supplementary foods because (1) root vegetables in the rumen lacked the plant parts that are removed by harvesting machinery; (2) to our knowledge, moose have never been observed to dig up root vegetables from the soil to eat; (3) the only way for moose to get access to silage is through supplementary feeding, and (4) there is independent evidence that land owners commonly place root vegetables and grass silage at supplementary feeding stations in southern Sweden^28^. *Malus domestica* (apple) was also found in a few samples. Whereas apples are used as supplementary feeds they can also be accessed by moose directly from trees. Therefore we treat apples separately from supplementary foods.

The proportion of rumen samples in which plant categories occurred also indicates a plant’s relative importance in the moose diet for this region (Fig. 1SI Supplementary Information). Almost every sampled individual (92%) had eaten *P. sylvestris* and 75–85% of individuals had eaten the three dwarf bushes. Mean diet composition for each subpopulation is presented in Table 2SI (Supplementary Information).

### Moose body mass and reproductive performance

**Figure 3:**
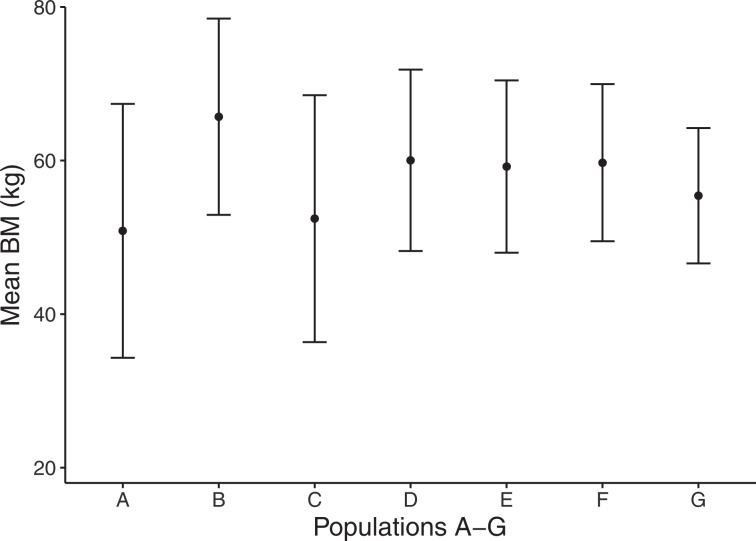
Mean (±SD) carcass body mass (BM) for N = 222 calves (6–9 months old at sampling) of seven moose populations in Southern Sweden, Oct 2014-Feb 2015. For names of populations (moose management areas), see Fig. 1.

The mean body mass (BM) of moose calves across all study areas was 58 ± 1 SE kg (N = 222). Using data reported by hunters to national data bases for the populations in our study, we found that there was a positive relationship between the 5-year average (2012–2016) mean calf BM and the equivalent mean number of observed calves per cow moose (Pearson correlation = 0.863, p = 0.012). There was also a significant positive correlation between mean calf BM and proportion of females observed with calf or calves (Pearson correlation = 0.839, p = 0.018). Therefore calf BM appeared to be a suitable indicator of population performance in our study. There was a significant difference between the seven populations in terms of mean calf BM (ANOVA, F = 3.58; p = 0.002; Fig. 3). However, when subpopulations (N = 22) were nested within populations in the model, the difference was not significant at the 5% level (nested ANOVA, Z = 1.47, p = 0.07). This indicates high variation within populations.

### Relationship between calf body mass and diet composition

**Figure 4:**
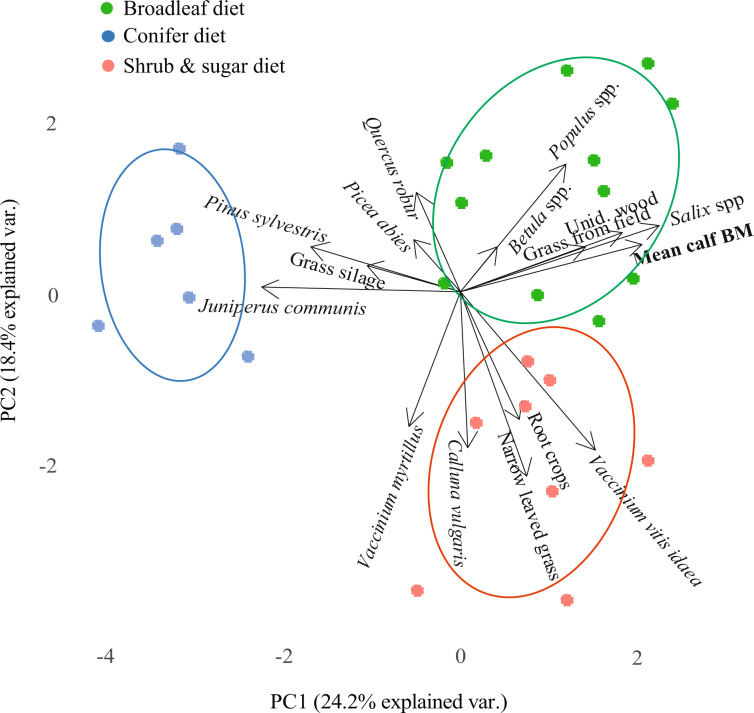
Bi-plot combining loadings and scores from Principal Component Analysis (PCA) of the mean diet compositions of 26 moose subpopulations (moose management units, MMU) in southern Sweden, sampled during winter. For each subpopulation (dots) the percentage of dry matter per plant category (15 categories) identified by macroscopy of rumen samples (tot N = 252) is included in the model, as is the mean calf BM. The pattern indicates that one cluster (“the broadleaf diet”, green) is associated with relatively high calf BM, while another cluster (“the conifer diet”, blue) is associated with low calf BM on PC1, and a third cluster (“the shrubs and sugar diet”, red) is associated with intermediate calf BM on PC2. Ellipses, drawn retrospectively, illustrate 95% confidence limits for each cluster.

The two first components of the principal component analysis (PCA) that we used to assess the 14 most dominant plant categories in the rumen samples (in terms of % dm), root vegetables, and mean calf BM of the 26 subpopulations (for which we had ≥5 individual rumen samples with macroscopy results and therefore included in the PCA, see Methods), together explained 43% of the variation in the data set (Table 3SI Supplementary Information). The scores plotted on the two axes showed three distinct diet clusters (Fig. 4). The plant categories that were most strongly correlated in a cluster with high mean calf BM (upper right corner of the plot, see positive values for PC1 in Table 3SI Supplementary Information) were *Salix* spp., unidentified wood, *V. vitis-idaea*, grass from field and *Populus* spp.. Due to the relatively high percentage of broadleaves in this diet cluster compared to the other two, we call this cluster the “broadleaf diet” for descriptive purposes (even though more than just broadleaved trees are included).

The plant categories that most strongly correlated with lowest mean calf BM (left side of the plot, see negative values for PC1 in Table 3SI Supplementary Information), were *Juniper communis* and *P. sylvestris* (Fig. 4). Although both conifers were present in the diet of all seven populations, their relative proportions were substantially higher in the six subpopulations within this cluster of lowest mean calf BM (15 times more *J. communis* and twice as much *P. sylvestris* on average; Table 2SI Supplementary Information) compared to the other subpopulations. We call this diet cluster the “conifer diet”. The mean proportion of *Salix* was approximately ten times higher in the 16 subpopulations of the broadleaf diet cluster (1.4–30.5%, mean 13.4% dm; Table 2SI Supplementary Information), than in the six subpopulations of the conifer diet cluster (0–2.6% *Salix*, mean 1.2% dm). The only broadleaved tree that was present in the “conifer diet” at higher proportions than in the “broadleaf diet” was *Q. robur*. However, *Q. robur* did not contribute strongly to PC1, likely because only two subpopulations had relatively high proportions of this tree species in their rumens. Three of the six subpopulations in the “conifer diet” cluster originated from moose management area A, the only study area from which we obtained more samples from late (Dec-Jan) than early (Oct-Nov) winter (Table 1SI). Hypothetically, this could have inflated the proportion of conifer in the associated data. However, the mean % of conifer (*P. sylvestris* + *P. abies* + *J. communis*) in late winter samples from area A was lower (53%, N = 18) than in early winter (63%, N = 8), ruling out the imbalance in collection dates as an explanation.

The third diet cluster we call the “shrubs and sugar diet” (lower part of the plot, see negative values for PC2 in Table 3SI Supplementary Information). This diet was characterised by similar diversity as the “broadleaf diet” (Table 1), but had higher occurrence of shrubs and sugar/starch-rich root crops (Fig. 4). The variables with the highest values in this cluster were twigs from the shrubs *V. vitis idaea, C. vulgaris* and *V. myrtillus*, narrow-leaved grass and root vegetables (Table 3SI Supplementary Information). Three of eight subpopulations in the cluster belonged to population G, a population with intermediate calf BM (Fig. 3, Table 4SI Supplementary Information). In this population 30% of individuals had root vegetables in their rumen: *B. vulgaris* 17% (of individuals) and *D. carota* 13%. These foods represented in average 8% dry matter of their diet (ranging between 4–14% dm among subpopulation means). None of the other five subpopulations in this cluster had root vegetables in their diet.

**Table 1:** The number of identified plant categories (PCat) in total and mean per sample, and the number of unique plant categories and proportion of exclusive plant categories found in rumen samples by macroscopy for each of the seven moose populations (MMA) included in this study.

Diet type, MMA	Mean calf BM	N	% low-weight	PCat	Mean PCat/ sample	Nb unique PCat	% Excl PCat	Name of unique PCat (*n*)
“Broadleaf diet”								
B	64.8	47	4.5	32	8.2	4	9%	*Avena* sp. (1) *S. tuberosum* (1) *C. avellana* (1) *F. sylvatica* (1)
D	60.0	67	25.9	29	8.1	1	2%	*Larix* sp. (1)
E	59.8	42	19.4	27	8.5	2	5%	*S. aucuparia* (1) *Rosa* sp. (1)
F	59.7	29	16.7	22	6.8	1	2%	*L. xylosteum* (1)
“Shrubs and sugar diet”								
G	54.9	46	40.0	29	8.6	3	7%	*V. oxycoccus* (1) Apiaceae flower (1) *D. carota* (6)
“Conifer diet”								
C	52.3	36	40.9	22	6.8	0	0%	
A	51.6	35	47.4	25	6.2	0	0%	
**All**		**302**		**44**		**11**		

The MMAs are sorted according to their mean calf body mass (BM, in kg) and diet type according to Fig. 4. The names of the unique plant categories are listed, indicating (in bracket) how many rumen samples they were found in. The proportion exclusive plant categories are defined as plant categories not found elsewhere as a percentage of the study total (44 plant categories). N = number of rumen samples (moose of all ages). Also listed is the percent of low-weight calves, i.e. calves with BM < 0.5 SD from the mean BM across all populations (i.e. < 51.9 kg dressed carcass).

**Figure 5:**
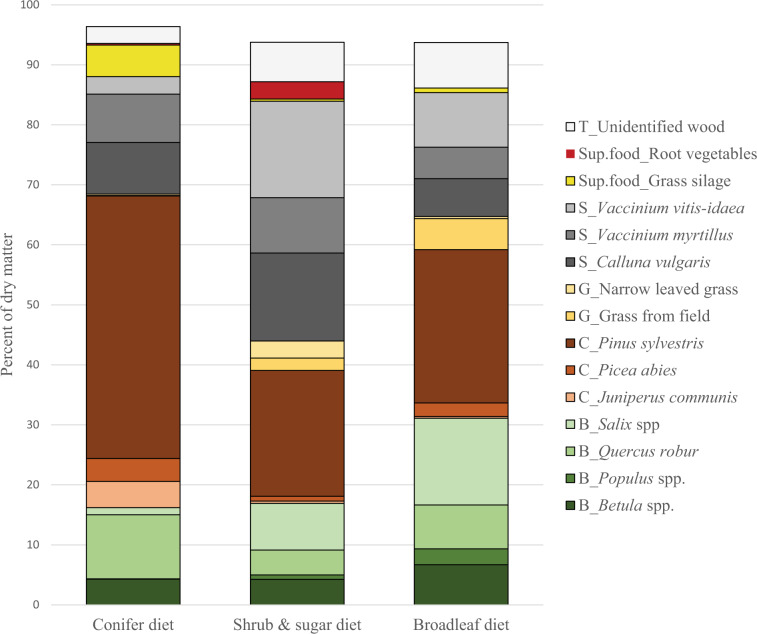
Diet composition of three distinct moose winter diet types, southern Sweden. The types were determined by principal components analysis of 252 individual rumen samples averaged at the level of the 26 subpopulations (Fig. 3). Displayed are the proportions of the fifteen plant categories included in the PCA, per diet type. Plant categories are sorted into classes: B = broadleaved trees; C = conifer trees; G = grass; S = shrubs; T = trees; Sup. food = supplementary food (i.e. deliberately placed into the environment by people as food for wild animals).

Agricultural crops, likely from supplementary feeding, were also found in 7 other subpopulations not having the “shrubs and sugar diet” (of 26). Grass silage was found predominantly in population A (the “conifer diet”), which had the lowest mean calf BM (3 subpopulations included). In population A, we registered 27% of individuals with silage in their rumens, representing in average 11% of dm (5–27% among subpopulation means). Of the subpopulations in the PCA that belonged to the four moose populations (B, D, E and F) with mean calf BM of ≥60 kg, only 3–5% (mean 4%) of the individuals had grass silage in their rumen (none had root vegetables). In those areas grass silage occurred at 0–6% of dm. However, not all the populations with the lowest BM had more supplementary food in their diet: in the population with the second lowest mean calf BM, C (having the “conifer diet” like population A, but without grass silage), only 6% of individuals sampled had eaten supplementary food according to macroscopy (*B. vulgaris*, 1.4% of dm). The fifteen plant categories included in the analysis (Table 3SI Supplementary Information) differed in their relative contribution to the moose winter diet among the three diet clusters, with *P. sylvestris* contributing the most in all clusters (Fig. 5).

### Relationship between calf body mass and diet diversity

**Figure 6:**
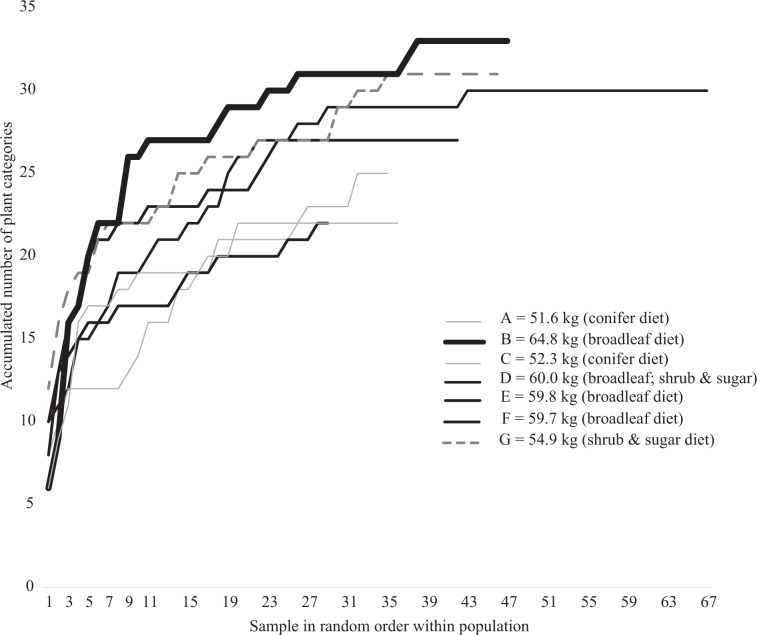
Sample-based species accumulation curves for the number of species in the diet of seven moose populations in southern Sweden. Species numbers from rumen samples (random order within population) analysed using macroscopy, collected between 23-Oct-2014 and 31-Jan-2015. Mean calf BM per population is illustrated by the thickness of the curve. Colours indicate which diet type the population predominantly represents. For names of MMA, see Fig. 1.

There was a positive correlation between calf BM and the number of plant categories found per rumen sample (Table 1), at both the population level (Pearson correlation 0.583, p = 0.002) and subpopulation level (Pearson correlation 0.387, p = 0.05). In the sample-based species accumulation curves a high trajectory illustrates a high accumulated number of plant categories among samples within a moose population. Our results indicated that the moose population with the highest mean calf BM had the steepest of the high trajectories (Fig. 6). This indicates that a higher proportion of individuals in this population had rumen samples that were more diverse. Only one population with relatively low BM (population G) had a high trajectory. This population, which belongs to the cluster (Fig. 4) we call the “shrubs and sugar diet”, had the highest mean number of plant categories per sample of all populations (Table 1). The two populations with the lowest mean calf BM (A and C), representing the “conifer diet”, had zero ‘exclusive’ plant categories (i.e. these populations had no plant categories that did not occur in other areas, see Methods). Moose belonging to the southernmost study area G with the “shrubs and sugar diet”, had a relatively high proportion of exclusive plant categories.

Due to a significant (positive) covariation between the % dm of *Salix* spp (the broadleaf tree that is most strongly associated with calf BM) and the mean number of plant categories per rumen sample per subpopulation (Pearson correlation 0.392, p = 0.048), we could not separate the relative effects of diet diversity and dietary proportion of broadleaved trees on calf BM.

### Relationship between calf body mass and landscape scale habitat variables (food availability)

Of the four habitat variables included in our multivariate tests of the landscape analysis, proportion of forest being young (our index of browse availability, see Methods) was consistently a significant part of all candidate models for both response variables (subpopulation mean calf BM and likelihood for low-weight calves) (Table 2, Table 5SI Supplementary Information). The mean proportion of young forest across subpopulations was 16%. Doubling this proportion (an arbitrarily chosen increase to indicate the potential effect) would correspond to an increase in calf BM of approximately 11 kg, based on the most parsimonious nested model (Table 2). Similarly, a doubling of young forest would reduce the likelihood of low-weight calves from 25% to 5%. There was also a higher likelihood of calves being low-weight with increasing proportion of area being forest (forest of all ages), but this was strongly countered by proportion of young forest of all forest land (Fig. 2SI Supplementary Information). The mean proportion of forest across subpopulations was 70%. Halving this proportion (also an arbitrary change to indicate the effect) would reduce the likelihood of calves being low-weight from approximately 25% to 8%.

**Table 2:** Multivariate modelling relating landscape scale habitat variables to A) mean body mass of moose calves (BM, dressed carcass kg); and B) the likelihood of these calves having particularly low BM.

*The most parsimonious nested model*					
A) Response = calf body mass (kg)	β	SE	df	t value	p-value
(Intercept)^a^	46.7	5.2	183	9.0	0.000
% of forest being young	68.5	31.1	30	2.2	0.036
**B) Response = low-weight calves**	**β**	**SE**		**z value**	**p-value**
(Intercept)^b^	−1.9	1.3		−1.4	0.164
% forest of total area	3.9	1.8		2.1	0.033
% of forest being young	−12.1	4.2		−2.9	0.004

Individuals (N = 222, harvested in Southern Sweden, Oct 2014-Feb 2015) were classified as low-weight if BM was at least 0.5 standard deviation below the mean BM of all calves: <51.9 kg. Forest data were collected from GIS data of a circular area with r = 10 km from the centre of each subpopulation. In the models, subpopulations were nested with population as a random intercept effect. Variables presented are those remaining after reducing collinearity and using stepwise inclusion and exclusion of variables, upon which the most parsimonious model was selected by AIC (AIC values and more candidate models, including non-nested models, are given in Table 5SI).

^a^mixed model with subpopulation (N = 39) nested in population (N = 7) as random intercept. Model selection (comparing models with different fixed, but the same random effects) was done with ML fitting, but the final model is presented with REML as this is considered to give more precise estimates of the coefficients.

^b^logit regression, binomial, with subpopulation (N = 39) nested in population (N = 7) as random intercept.

## Discussion and Conclusion

By studying a large number of individual moose inhabiting over ten thousand km^2^ of highly modified rural landscape, we found that differences in plant diversity and composition of the winter diet could in part account for variation in moose calf body mass. Dietary diversity (i.e. number of plant categories in the rumen sample) was positively associated with calf body mass. The diet composition formed three distinct clusters with calf body mass. We call these diet types the “conifer diet” (low body mass), the “shrubs and sugar diet” (intermediate body mass), and the “broadleaf diet” (higher body mass). We also found that mean calf body mass increased, and the proportion of calves with low body mass in subpopulations decreased, with higher availability of young forest in the landscape. This phase of the forest rotation period (ca 0–15 yrs since clear cutting and regeneration) represents the habitat type with the highest concentrations of tree browse within browsing height^7,29^. Our results thus support the hypothesis^30^ that variation in landscape scale winter food composition and quantity is one explanatory factor behind regional differences in body mass in Scandinavian moose^31^. This result has wide implications, as body conditions early in life can affect many aspects of adulthood, creating long-lasting effects on demographics^22^. It may be such inter-generational relationships that underlie the significant correlation in our study populations between mean calf body mass and estimates of calf production by adult females as in^24^.

Our results add landscape-level credence to previous observations that diet mixing is important for generalist herbivores^32–34^. More specifically, in our study we were able to identify three distinct types of diet mixtures (Fig. 4). The “broadleaf diet” was associated with relatively high mean calf body mass. The identified plant categories most strongly correlated with higher mean calf body mass were *Salix* spp., *V. vitis-idaea, Populus* spp., and grass from fields. These findings are consistent with other studies in Sweden that identified plants of the genera *Salix* and *Populus* to be highly selected for by moose during winter^35,36^. Furthermore, *Salix* spp. twigs appear to be nutritionally well balanced food items for this herbivore^37^. Notably, the six subpopulations with the lowest mean calf body mass had one tenth the proportion of *Salix* spp. in their diet compared to the subpopulations in the broadleaf diet cluster (Table 2SI).

A low amount of broadleaved tree species, limited plant diversity, and a lack of exclusive plants, signified the “conifer diet”, which was in-turn associated with relatively low subpopulation mean calf body mass. The six subpopulations with the lowest mean calf body mass in the study (all included in the “conifer diet”) had twice as high proportion of *P. sylvestris* in their diet, and 15 times more *J. communis* than the other subpopulations. This does not mean that these two food plants, commonly reported as moose winter forage^35,36,38^, are bad for moose. Instead, our results support the idea of complementarity^19,33,39^. Needles from conifers, and twigs from broadleaved trees and dwarf bushes, complement each other nutritionally, as they differ in their concentrations of dietary fibres, easily digestible carbohydrates and protein (Felton, A. unpublished data), and likely also in secondary metabolites. When herbivores have the opportunity to mix such complementary items, they are more likely to reach their nutritional target^37^, while potentially avoiding excessive doses of plant defence chemicals^12^. Repeated large doses of a limited number of plant items may hamper the animal’s ability to balance their nutrient intake, with negative effects on their fitness^40^.

The third diet type, the “shrubs and sugar diet”, largely resembled the diversity and composition of the “broadleaf diet”, but had more shrubs and sugar/starch-rich agricultural crops, and less broadleaved trees. The subpopulations consuming this diet had intermediate mean calf body mass. This diet type was relatively rich in plant categories, including root crops such as beets and carrots. These crops are common supplementary foods in the region^28^, meaning that the root crops (stripped of leaves) are deliberately placed into the environment with the aim of regulating the availability of food for the wild animals^41^. These root crops have been cultivated for enhanced energy content and have nutritional compositions that are highly inconsistent with the natural winter diet of moose^30^. Large intakes of such items can increase the risk for ruminal acidosis^42^, and teeth deterioration, if items are high in sucrose (e.g. beets)^43^. Furthermore, although these crops are rich in digestible macronutrients, the inclusion of cultivated plants in large doses in cervid diets does not necessarily reduce the dietary importance of natural food plants^44^.

Concerns regarding supplementary feeding are not limited to Sweden^30^, as these practices have been associated with adverse impacts on the health^41^ and ecology^45^ of free-ranging ungulates internationally, even though positive effects of supplementary feeding on ungulate survival and reproduction also have been observed^45^. Due to covariance in our data-set between the intake of supplementary foods with diet diversity and ungulate population density, a clear interpretation of these particular linkages is not possible in our study. It also remains to be determined whether the high proportion of shrubs in this diet is making a positive, neutral, or negative contribution.

Positive correlations between the presence of certain plant species in rumens and variation in body mass, found in this study and others^22,46^, depend on the availability of these plants in the landscape. The availability of many of the moose’ key food resources is strongly influenced by the amount of forest in young succession stages^7,15^. A direct association between this factor and moose population performance has also been established for southern Norway^31^. It was therefore not surprising that we found a higher likelihood of a moose subpopulation having individuals of very low body mass, as well as the calf body mass *per se* being lower, if the proportion of young forest was lower.

Interestingly, we found that the higher the proportion of forest (i.e. forests of all ages), the greater the likelihood of having many low-weight calves. This may reflect the importance of landscape diversity. The forest in our study areas consisted of predominantly production forests^47^. Areas dominated by production forests may have a lower diversity of other habitat types (including fewer “edge” areas often preferred by moose^48^), and therefore a lower diversity of available forage plants. As the proportion of forest cover declines, other land uses (e.g. crop lands, grazing land, fallows) are likely to vary in their influence on moose BM. At too low a forest cover, however, the absolute availability of young forest will correspondingly decline, with a negative associated impact on moose BM. The lack of protective cover *per se* may also be negative for these animals, as they select for forest cover due to predation risk and adverse weather conditions^49–51^.

Any negative impact of poor food availability on the body mass of moose can have compounding interactions with other environmental stressors, because individuals are less likely to be buffered against environmental stochasticity^6^. Further research spanning multiple years would be needed to detect such compounding interactions. Because our rumen analysis results show that plant diversity can be an important dietary factor explaining variation in moose calf body mass, and because moose are known to select for heterogeneous environments^52^, we recommend that future analyses estimate finer scale variation in land use.

We emphasize that in addition to the dietary factors considered in this study, many other variables can be expected to influence moose calf body mass. Such variables include, for example, the previous years’ weather, and the age and body reserves of the mother^26,53^. Furthermore, there could be differences in hunting regimes among moose management areas and units. Further research is needed to assess the relative importance of these drivers which we did not account for in this study. For example, to decipher the causal processes that underlie variation in diet and body mass of moose, a controlled experiment, on a smaller spatial scale, with manipulated quality and quantity of forage would be necessary. We also emphasize that because moose use body stores built up during the growing season to meet the demands of the northern winter^19^, the composition of their spring and summer diet should strongly influence the body mass values we observe during winter time. We speculate that the relationship we have identified here between calf body mass and winter diet composition also reflects the habitat quality these animals experience during the growing season, both in terms of diet composition/diversity and relative forage quantity. This is supported by the fact that the proportion of forest land comprised of regenerating forest is a strong predictor of ungulate forage biomass during the growing season^15^.

Because we found that moose diets higher in plant diversity were associated with higher mean calf body mass, we suggest that efforts to increase the diversity and availability of different food plants – trees, bushes and herbaceous vegetation – within both forest and agricultural landscapes would likely benefit moose populations and potentially other cervids. Importantly, not all plant items that contribute to dietary diversity have an equal value. For example, attempts to increase dietary diversity via supplementary feeding entails many caveats due to a number of potential adverse implications^30^. This caveat is exemplified by the “shrubs and sugar diet” in our study. In contrast, our results emphasize the disproportionate importance of increasing the availability of broadleaves, and the genera *Salix* and *Populus* in particular, to benefit moose.

To achieve a greater diversity and availability of food plants, forest managers could work with improving light availability to the forest floor in mature stands, either by adjusting stem density or the composition of the production tree species. For example, converting a stand from Norway spruce to Scot’s pine creates environments with more understory light, even when timber volumes remain equal^54^. Not only would such efforts directly increase the availability of tree-sourced food resources, but the higher light availability provided in the understory of intermediate to older stands should increase the coverage of understory dwarf shrubs (e.g. *V. myrtillus* and *V. vitis-idaea*) that are a common food resource for many cervids^21,38,55,56, this study^. The cover of these shrub species has decreased in Sweden since national scale measurements began in 1985^57^, and their declines are thought to mainly be driven by forestry practices resulting in poor light availability in older conifer production stands^58,59^. Notably, these trends are not isolated to Sweden. For example, in the closed forests of western North America the loss of shade-intolerant plant species in the understory is accelerating the loss of body fat in lactating deer (*Cervus elaphus*)^60^.

There may also be associated benefits for forestry from a more diverse forest landscape. For example, increasing the prevalence of suitable food resources in different parts of the landscape may reduce the concentration and intensity of browsing damage in young production stands^15,61^. The risk of not doing so is that positive feedback loops could develop whereby forest owners plant larger areas with unpalatable production tree species like Norway spruce, which in-turn concentrates browsing pressure on remaining stands of more palatable tree species, thereby favouring the further establishment of production stands with Norway spruce.

In conclusion, our results emphasize the importance of diet mixing for a large herbivore inhabiting highly modified landscapes. Furthermore, our results highlight the importance of taking both plant diversity and food quantity into account when assessing the effect of landscape modifications on large herbivores. In highly modified landscapes, efforts to increase both the abundance and the diversity of plants within the forest and agricultural matrix should benefit populations of large herbivore species. These findings are consistent with current international discussions regarding the potential benefits of adopting more diversified approaches to production forestry^62^. Incentives to do so include the associated benefits to biodiversity, the increased adaptive capacity, resilience and the breadth of ecosystem goods and services provided by forests^63,64^.

## Methods and Materials

### Study areas

The study areas are in the hemiboreal climate zone of southern Sweden and cover over 10 000 km^2^. The mean annual precipitation is 700 mm with 25–100 days of annual snow cover. Sixty-three percent of the terrestrial area of this region is forested^65^, of which 16% is set aside from timber harvesting due to conservation purposes or low forest productivity^47^. The vast majority of the remaining forest area (>80%) is used for forestry. Norway spruce (*Picea abies*) or Scots pine (*Pinus sylvestris*) production forests dominate forest cover, sometimes mixed with naturally regenerated broadleaves, primarily birch, (*Betula pubescens*, *B. pendula*), rowan (*Sorbus aucuparia*), aspen (*Populus tremula*) and oak (*Quercus robur*). Forest land is usually subject to scarification prior to regeneration, which favours deciduous recruitment and forage production in young stands as well as the establishment and growth of coniferous plants. The study region is divided into moose management areas (MMA) thought to support more or less distinct moose populations due to barriers such as fenced highways and major water bodies^66^. We use the term ‘population’ accordingly. Moose management areas are divided into moose management units (MMU), within which the annual hunt occurs. We refer to moose belonging to one MMU as a ‘subpopulation’. We obtained samples from harvested moose from 55 MMUs within seven MMAs (Fig. 1). Hunting statistics indicate that the seven MMAs differ in moose population density (range 0.22–0.41 moose harvested km^−2^ in 2014/15; Fig. 1). As our focus was the moose winter diet, we collected samples starting at 23-Oct-2014. By this time, most deciduous trees had shed their leaves or largely lacked chlorophyll, and trial checks of rumen contents lacked green leaves.

### Collection of rumen samples, body mass data and age analysis

Volunteer hunting teams within each MMU provided samples from moose harvested as part of the yearly hunt, not biased towards diseased individuals. Hunters were instructed how to collect samples (Supplementary Information). Hunters noted the sex, date, location, and carcass BM (after skin, head, blood, metapodials and internal organs removed; hereafter referred to BM). Rumen samples were collected immediately after harvest. Lower jaws were collected for age analysis (except from calves; age classes defined in Supplementary Information). All samples were frozen at −20 °C shortly after sampling. To estimate age we sectioned one first-molar tooth and counted the cementum layer^67^. Rumen samples, jaws and BM data from 447 moose (426 from the seven MMAs and 21 from adjacent areas) were obtained between 23-Oct-2014 and 22-Feb-2015 (Table 1SI). About 65% of samples stemmed from Oct-Nov when snow-cover was absent or very sparse in most of the region. Sampling date distribution was similar across MMAs (Table 1SI), with the ratio of samples obtained in early (Oct–Nov) versus late (Dec–Jan) winter being approximately 70:30. An exception was area A where proportions were 30:70. The proportion of calves varied between MMAs (38–80%, Table 1SI).

### Estimates of diet composition (macroscopy)

Due to prohibitive cost, we analysed 323 out of 447 available rumen samples for plant composition using macroscopy. We prioritized samples with complete information regarding sex, age and BM. Frozen rumen samples were defrosted, mixed, and analysed through macroscopic analysis to identify plant fragments to the lowest taxonomic level possible (hereafter “plant categories”), following a standard method (Supplementary Information)^56^. Grasses were differentiated into four categories: those with narrow leaves, those with broad leaves and originating from forest (hereon referred to as “forest grass”), those with broad leaves and originating from field (“grass from fields”), and fermented grass (“grass silage”). After drying, the proportion of each food item per sample was calculated. Twenty-one of the 323 samples with macroscopy results came from outside the main study populations. They are included in the overall mean diet composition of the study, but not in statistical analyses linking diets to BM.

### Estimates of diet diversity

First, we calculated the mean number of plant categories per rumen sample per subpopulation, to test correlations with mean subpopulation calf BM. Second, we plotted sample-based species accumulation curves^68^ for the seven moose populations, based on individual rumen samples in random order, to assess how sample size influenced patterns of plant category richness and its relation to mean calf BM. Samples collected between 23-Oct and 31-Jan were included, to avoid bias from occasional samples obtained during February. Third, we calculated the proportion of ‘exclusive’ plant categories found in single populations^69^. Exclusive categories are those not found elsewhere, presented as a percentage of the study’s total number of identified plant categories.

### Moose BM, proportion of low-weight individuals and calf production

From the volunteer MMU hunters’ data, we quantified the proportion of low-weight calves in each population as an indicator of poor condition. ‘Low-weight’ was defined by a BM less than 0.5 SD below mean BM for calves across all populations (i.e. <51.9 kg). We used this indicator in our landscape scale analysis of the influence of food availability on calf BM (see below). To establish whether calf BM reflects population reproductive status, we tested the correlation between population mean calf BM (N = 7) and two estimates of reproduction recorded routinely by Swedish hunters^70^: mean observed number of calves per female, and proportion of females observed with at least one calf (Table 4SI Supplementary Information). We calculated the 5-year average (2012/13–2016/17) for the three variables per population. In the Swedish game management system, a minimum of 5000 observation hours is recommended to make statistical comparisons of the observation data^71^. While there were sufficient observation hours at the population level in our study, there were too few observation hours on the subpopulation level to allow similar analysis.

### Data on landscape scale habitat variables (food availability)

We inferred food availability at the subpopulation level from landscape scale habitat variables from the Swedish Environmental Protection Agency. Data were calculated within a 10 km radius (314 km^2^) from the centre of each subpopulation using Esri ArcGis software 10.4.1^72^. Area of different land use categories (Supplementary Information) was obtained with 25 × 25 m pixel resolution^73^. The area of forest cleared between 1998 and 2014 within the same radius was retrieved from the Swedish Forest Agency’s online data^74^. Because this phase of the forest rotation period (ca 0–15 yrs since clear cutting and planting/ natural regeneration) represents the habitat type with the highest concentrations of tree browse within browsing height^7,29^, we calculated the proportion of young forest of total forest area, and use it as an index of browse availability. Note that in Sweden, the use of herbicides is restricted on forest land^75^.

### Statistical analyses

Statistical analyses were conducted in R × 64, 3.4.3^76^. Visual data exploration and an outlier exclusion are described in Supplementary Information (Supplementary Methods). We calculated covariance matrix coefficients to assess whether results of subpopulation BM were spatially auto-correlated. Even though covariance correlations were fairly low (overall 0.08), we used nested analyses whenever possible in comparisons of subpopulations. We used Analysis of Variance (ANOVA) to assess if calf BM differed between the seven moose populations. Nested ANOVA was then used to compare mean calf BM among subpopulations. Only subpopulations with data from ≥5 calves were included (N = 22 out of 26). To establish whether calf BM indicates reproductive performance, we tested the population level correlation (Pearson) between the 5-year average of the calf BM, and the 5-year average of the two estimates of reproduction.

Because each individual rumen sample is a snap shot in time, whereas BM is a longer term reflection of their diet, we tested the relationship between diet and BM by combining all individuals in a subpopulation and using their mean diet composition. However, as a first step we tested whether it was justified to include rumen results from all age-sex classes in a subpopulation average. To do so we conducted a principal component analysis (PCA) (‘prcomp’ function in R), using log transformed, centred and scaled values, including individuals with known sex and age (N = 302) and a subset of the 44 plant categories identified. This subset included all plant categories that were represented with at least 1% of dm across all subpopulations (14 categories). In addition, because of our a priori interest in supplementary fed root vegetables (due to their suspected disproportionate effects on moose digestion and forage selection^30^), we also included within this subset “all root vegetables”, which surpassed the 1% threshold when *B. vulgaris, D. carota* and *S. tuberosum* were combined (0–14% of dm among subpopulations, mean 1% ± 0.6% SE). Together these fifteen plant categories represented 95% of dm. While any inclusion threshold (i.e 1% of dm) is to some extent subjective, in this case it enabled us to remove the noise of rare food items, increase the explanatory power of the model, while simultaneously capturing the dominating food items, including supplementary foods. Because demographic classes did not represent different clusters in diet composition (Fig. 3SI, Table 6SI Supplementary Information), we proceeded to calculate a subpopulation mean across all age-sex classes.

We then used a similar PCA as above to assess whether some particular diets were associated with subpopulation mean calf BM. To do so we used mean % dry matter of the same 15 plant categories as described above, calculated for each subpopulations for which we had ≥5 individual rumen samples with macroscopy results (N = 26 subpopulations). For each subpopulation, the mean % dry matter per plant category was included, as was the mean calf BM.

We used Pearson correlation analysis to test the relationship between mean calf BM per population and the mean number of plant categories identified per rumen sample in that population. The same analysis was done at the subpopulation level, using nationally reported data for BM instead of our own to maximise the use of our macroscopy data. To see whether we could separate the effects on calf BM of diet diversity versus proportion of broadleaved trees in the diet we used Pearson correlation to test the covariation between the mean number of plant categories per rumen sample per subpopulation and the % dm of *Salix* spp (the broadleaved tree species that according to our results was most strongly associated with calf BM).

After the first removal and sorting of covariates in the landscape scale analyses (Supplementary Information), we proceeded to test four variables (proportion of forest in MMU area, proportion of forest being young, and proportion of forest area being fast-growing respectively slow-growing broadleaves). The relationship between landscape scale habitat variables and mean BM of calves was tested with mixed models (‘lme’), first using MMU nested in MMA, and then only MMA, as a random intercept variable (following the protocol of^77^). We then used stepwise backwards and forwards selection (‘stepAIC’ with maximum likelihood (ML)) from the full model until we obtained the most parsimonious models. We checked whether model assumptions were violated by assessing patterns in plots of residuals against fitted values. We ran the final model with restricted maximum likelihood (REML)^77, p. 122^. We tested the likelihood of low-weight calves in MMU with logistic regressions (‘glmer’, binomial family, logit link function) on the same four landscape variables (with and without MMU nested in MMA) using a binary variable for weight of individual calves below or above the weight limit described. Like for calf BM, the models were compared with AIC. Assessment of residual plots indicated that assumptions were not violated.

## Supplementary information

Supplementary Information.

## Data Availability

The data sets generated during and/or analysed during the current study are available from the corresponding author upon request.
