# Supplementary material for: Varied diets, including broadleaved forage, are important for a large herbivore species inhabiting highly modified landscapes
Source: Sci Rep. 2020 Feb 5;10:1904. doi: 10.1038/s41598-020-58673-5 (PMC7002458; doi:10.1038/s41598-020-58673-5)
Supplement: Supplementary file 1 — Supplementary Information. [file 41598_2020_58673_MOESM1_ESM.pdf]

Felton, A. M., Holmström, E., Malmsten, J., Felton, A., Cromsigt, J.P.G.M., Edenius. L., Ericsson, G., Widemo, F., Wam, H.K. Varied diets, including broadleaved forage, are important for a large herbivore species inhabiting highly modified landscapes. Supplementary Appendix

SUPPLEMENTARY INFORMATION

Varied diets, including broadleaved forage, are important for a large herbivore species inhabiting highly modified landscapes

Authors:

Annika M. Felton*[1], Emma Holmström[1], Jonas Malmsten[2], Adam Felton[1], Joris P.G.M. Cromsigt[2,3], Lars Edenius[2], Göran Ericsson[2], Fredrik Widemo[2], Hilde K. Wam[4]

Affiliations:

[1] Southern Swedish Forest Research Centre, Swedish University of Agricultural Sciences, PO Box 49, SE-230 53 Alnarp, Sweden

[2] Department of Wildlife, Fish and Environmental Studies, Swedish University of Agricultural Sciences (SLU), Umeå, SE-901 83, Sweden

[3] Centre for African Conservation Ecology, Department of Zoology, Nelson Mandela University, PO Box 77000, Port Elizabeth, 6031, South Africa

[4] Division of Forestry and Forest Resources, NIBIO, PO Box 115, Ås, NO-1431, Norway

* Corresponding author: annika.felton@slu.se; ph: +46 40415177; (no fax)

Felton, A. M., Holmström, E., Malmsten, J., Felton, A., Cromsigt, J.P.G.M., Edenius. L., Ericsson, G., Widemo, F., Wam, H.K. Varied diets, including broadleaved forage, are important for a large herbivore species inhabiting highly modified landscapes. Supplementary Appendix

Supplementary Information

Supplementary Methods

Calves were in our study defined as individuals born the previous spring (6-9 months old at sampling); yearlings as individuals born one year before that; and adults as all individuals born ≥ 2.5 yrs before sampling. Volunteer hunting teams within each MMU provided samples from moose harvested as part of the yearly hunt. Samples were also taken opportunistically from moose killed in vehicle collisions within the study areas (7 individuals in total). Hunters were instructed how to collect the samples via an online instruction video which can be found here: https://www.youtube.com/watch?v=VjpES8BK1Qw. Hunters mixed the contents of the rumen to obtain a thorough sample (Cederlund *et al.* 1980), squeezed a handful to remove excess rumen liquid, and obtained enough of these contents to fill a 1 litre plastic airtight container.

Rumen samples were analysed through macroscopic analysis to identify plant fragments, following a method used in ungulate research (Nichols, Akesson & Kjellander 2016). Macroscopic identification required washing 250 ml through a 4mm and 2mm mesh sieve to separate the material into easy to work with size categories. The fragments retained in both sieves were identified to the lowest taxonomic level possible (hereafter “plant categories”) using a magnifying glass, a floristic guide (Mossberg & Stenberg 2003) and a reference collection (located at Viltteknik, Stubbvik Danbyholm, 61196 Jönåker, Sweden). Each plant category, as well as unidentified material, was placed in separate tubes and dried at 70° C for 72 h, and weighed to the nearest 0.01g. We calculated the proportion of each food item (% dry matter (dm)) found by macroscopy in each individual sample (summing results from the 2mm and 4mm fractions). To assess how consistently this method produced results from different sub-portions of an original rumen sample, the same procedure was repeated (by the same person) on an additional sub-portion from 16 of the individuals already analysed. The analyst did not know the original sample ID. We used a two-sample t-test to assess whether the relative proportions of plant categories (% dm) differed between the two subportions.

To establish whether calf BM reflects population reproductive status, we tested the correlation between population mean calf BM (N=7) and two estimates of reproduction recorded routinely by Swedish hunters (Morellet *et al.* 2011): mean observed number of calves per

female, and proportion of females observed with at least one calf . We extracted these data from the systematic hunter-reported statistics in the Swedish national online databases www.viltdata.se (for the observations) and www.algdata.se (for the carcass weights; Table 4SI) for five hunting seasons (2012/13 – 2016/17).

We inferred food availability at the subpopulation level from landscape scale habitat variables available in publically available data source from the Swedish Environmental protection agency. Data was calculated by GIS for a circular area, with a 10 km radius (314 km^2) from the centre point of each subpopulation, using Esri software ArcGis Desktop software 10.4.1 Area of land use (forest, agriculture, urban, water, mire) and forest type (within the land use "forest", see below), was retrieved from a raster layer with pixel resolution of 25×25 m (CadasterENVSweden 2015). Forest types were divided in coniferous or broadleaved forest, and further divided in Scots pine or Norway spruce forest, and slow- (mainly oak or beech) or fast-growing broadleaves (mainly birch and aspen).

Statistical Analyses

Statistical analyses were conducted in R ×64, 3.4.3 (RCoreTeam 2017). All variables were visually explored using Cleveland dotplots to detect outliers in variables, as well as boxplots and interaction plots for factors (Zuur, Ieno & Elphick 2010). We omitted one outlier for calf BM (96 kg carcass weight, which is physically not possible, assuming it was a yearling wrongly classified by hunters into calf). A first check of data (regression analysis) was made in order to detect bias on BM due to harvest date, which was not significant, and therefore ignored in further analyses.

We tested the potential correlation in the data set between the mean % dm of "unidentified wood" per moose population (n = 7) and mean calf body mass (BM) of the population using Pearson correlation. Potentially, a large share of wood fragments that could not be assigned to a genus or species in rumen samples could be related to a high browsing pressure in the area, as heavily browsed trees may offer moose twigs devoid of buds to a greater extent.

We wanted to see whether or not it is legitimate to include rumen results from all age-sex classes in a subpopulation average measure of diet composition. If age-sex classes differ, they should not be merged. We therefore conducted a principal component analysis (PCA), including individuals with known sex and age (n = 302) and a subset of the 44 plant categories identified. This subset included all plant categories that were represented with at

least 1% of dm across all subpopulations (14 categories). In addition, because of our a priori interest in supplementary fed root vegetables (due to their suspected disproportionate effects on moose digestion and forage selection[31]), we also included within this subset "all root vegetables", which surpassed the 1% threshold when *B. vulgaris, D. carota* and *S. tuberosum* were combined (0-14% of dm among subpopulations, mean 1% ± 0.6% SE). Together these fifteen plant categories represented 96% of dm. While any inclusion threshold (i.e 1% of dm) is to some extent subjective, in this case it enabled us to remove the noise of rare food items, increase the explanatory power of the model, while simultaneously capture the dominating food items, including supplementary foods. For each moose individual, the % dm per plant category was included, and its respective demographic class (calf = 1, yearling = 2, adult female = 3, adult male = 4).

The first removal and sorting of covariates in the landscape scale analyses was done using multipanel scatterplots when there was collinearity between covariates. For all correlations > |0.6| (Zuur et al. 2009), we kept the variable with the lowest sum of correlations across covariates. We did not include, for example, area of conifers because it was strongly correlated with total forest area. All subpopulations were included in the landscape scale analyses, irrespective of sample sizes of moose data.

Supplementary Results

There was no relationship between the mean % of unidentified wood in rumen samples and mean calf BM of the moose population (Pearson correlation = 0,464, P = 0,294, n = 7). There was a large overlap in plant identifications between the first and the second round of analysing 16 rumen samples with macroscopy. In 91% of cases, if a plant item was present or absent in one round of analysis, it was likewise present or absent in the second round. Eight novel plant items for the whole study were identified when the first and second round were compared. With one exception, these eight novel identifications represented items that were present in very small proportions of the sample's total dm, ranging from 0.07% to 0.96%. The exception was "unidentified bark" which was found in 2 of the original samples (both ca 2% dm) but not at all in the second round. There was no significant difference in the relative proportions of plant categories present in the two rounds (t-value = -0.19, DF = 65, p = 0.846). Regarding the ten most dominant plant categories, the same general diet pattern was obtained from both assessment rounds (Fig. 4SI). Only % dm of *M. domestica* (apple)

differed considerably. We believe this had little bearing on our findings, because apples were found in only 5% of the rumen samples.

Principle component analysis of macroscopy results of 302 individual rumen samples indicated that individuals are representative of their area's overall diet regardless of their age or sex. First, the four different age-sex classes did not form distinct clusters in terms of % dm of different plant categories. The great majority of individuals fall within the main cluster of data points (Fig. 3SI). Of the 11 data points that stick out from the main cloud of data, 54% were calves, which is similar to their proportion in all samples (53%), which indicates that age class did not influence the pattern in diet composition. Only 22% of the total variation was explained by the first two components in this PCA. Second, the variable "age-sex class" (1-4) did not obtain high values in any of the principal components (Table 6SI). In the following analyses, all age-sex categories were therefore merged.

Felton, A. M., Holmström, E., Malmsten, J., Felton, A., Cromsigt, J.P.G.M., Edenius. L., Ericsson, G., Widemo, F., Wam, H.K. Varied diets, including broadleaved forage, are important for a large herbivore species inhabiting highly modified landscapes. Supplementary Appendix

Supplementary Figures

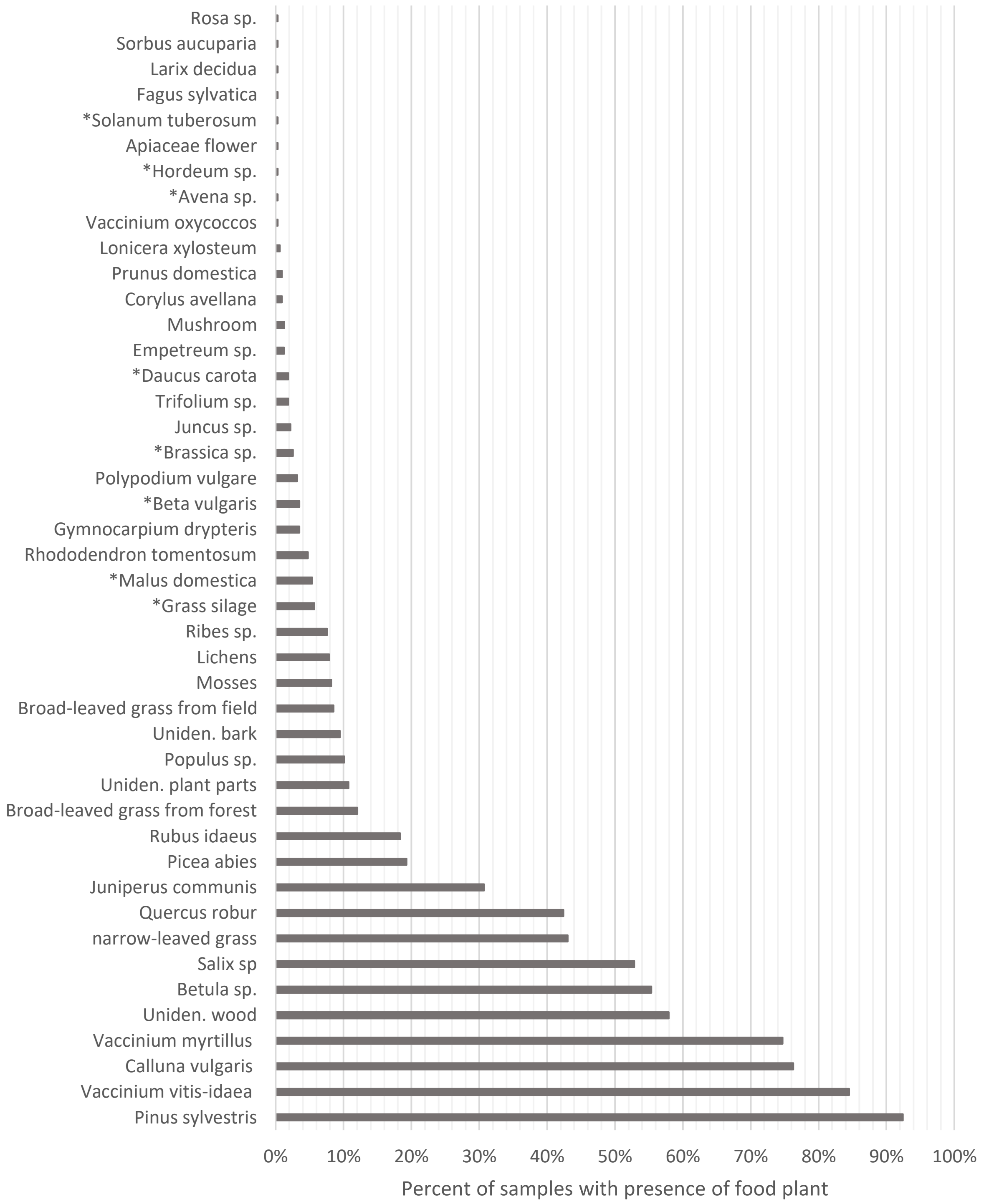

Figure 1SI. Percent of moose rumen samples, analysed with macroscopy, with presence of 44 different plant categories. Results are shown from 323 moose shot between 23-Oct-2014 and 22-Feb-2015 in southern Sweden. Cultivated crops are indicated with an asterix.

Felton, A. M., Holmström, E., Malmsten, J., Felton, A., Cromsigt, J.P.G.M., Edenius. L., Ericsson, G., Widemo, F., Wam, H.K. Varied diets, including broadleaved forage, are important for a large herbivore species inhabiting highly modified landscapes. Supplementary Appendix

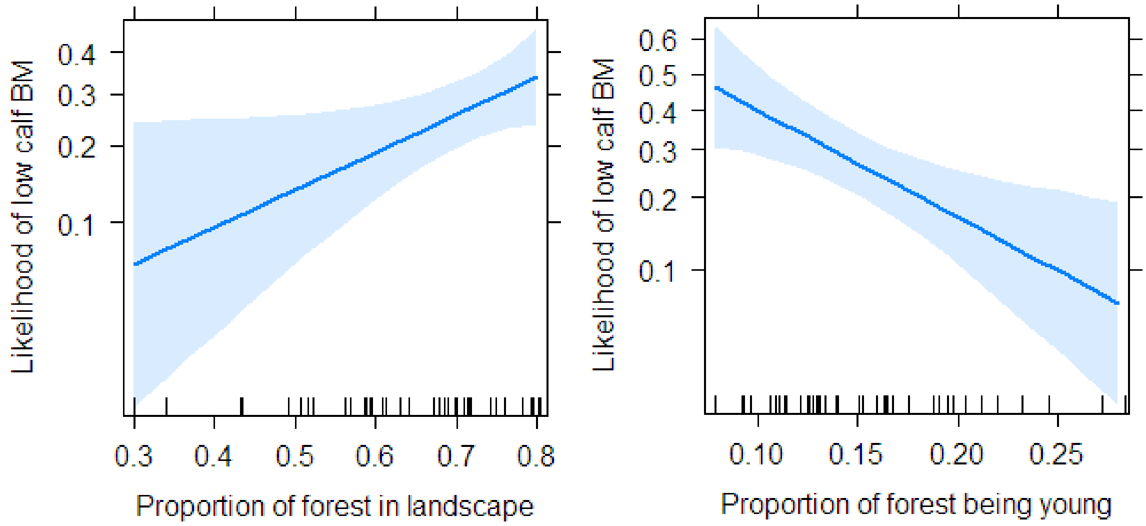

Figure 2SI. Linear fit (effect plots with 95% confidence bands) of the most parsimonious model of association between likelihood of moose subpopulations having calves with low body mass and **a)** proportion of forest in the landscape and **b)** the proportion of forest being young. Low BM was defined as < 0.5 SD from the mean BM across subpopulations (i.e. <51.9 kg dressed carcass). BM data from N= 222 calves (6-9 months old) in Southern Sweden, Oct 2014-Feb 2015. Forest data was collected from GIS data of a circular area with r=10 km from the centre point of the habitat range of each subpopulation.

Felton, A. M., Holmström, E., Malmsten, J., Felton, A., Cromsigt, J.P.G.M., Edenius. L., Ericsson, G., Widemo, F., Wam, H.K. Varied diets, including broadleaved forage, are important for a large herbivore species inhabiting highly modified landscapes. Supplementary Appendix

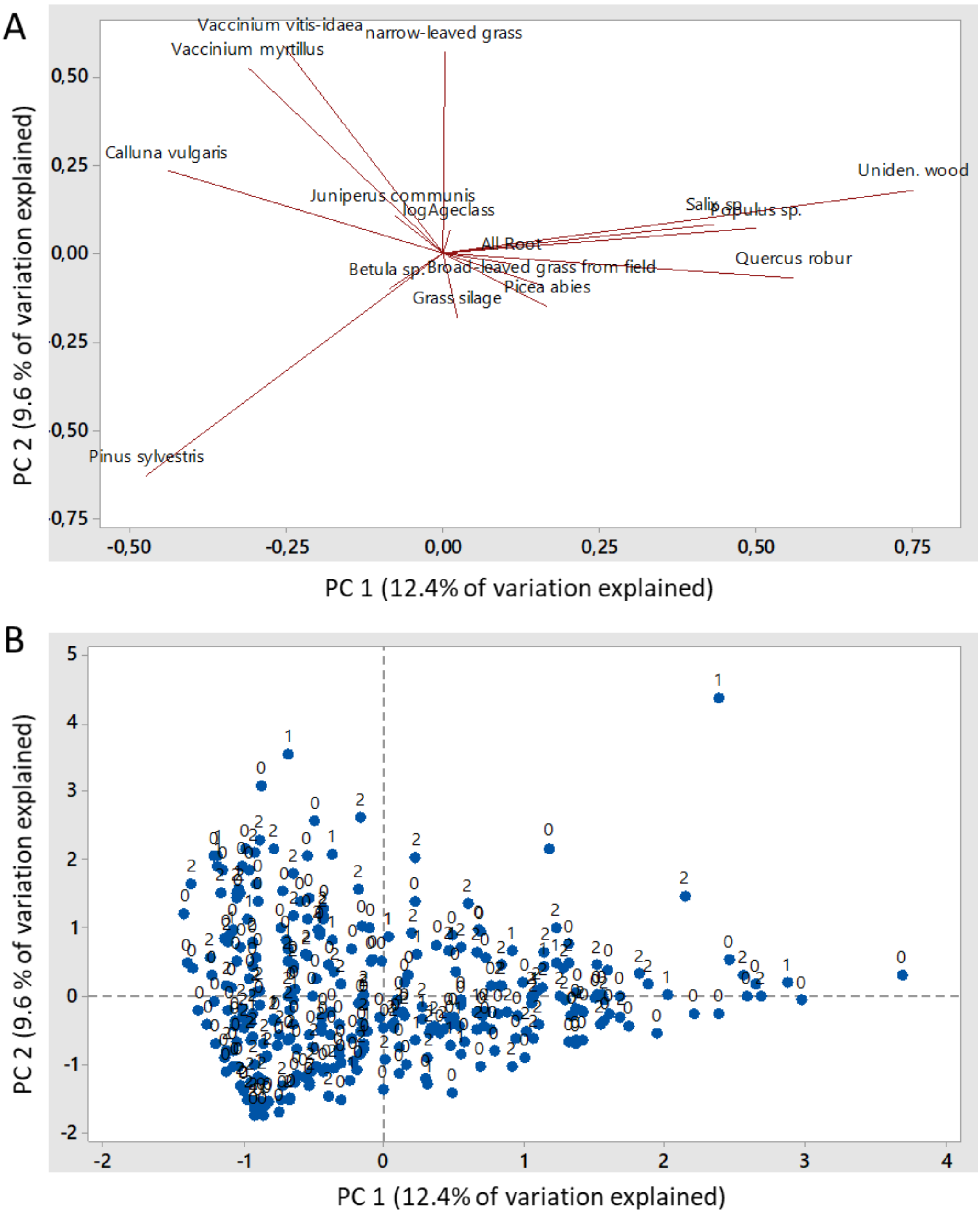

Figure 3SI. A) Loading plot from Principal Component Analysis (PCA) of the diet compositions of 302 moose individuals. For each individual rumen sample the percentage of dry matter per plant category (15 categories) identified by macroscopy is included in the model, as is the code of their respective age-sex class. B) Score plot from the same PCA showing that the age-sex categories of the individuals (calf = 1, yearling = 2, adult female = 3, adult male = 4) were evenly distributed along the loadings. Of the 11 data points that stick out from the main cloud of data, 54% were calves, which is similar to their proportion in all samples (53%).

Felton, A. M., Holmström, E., Malmsten, J., Felton, A., Cromsigt, J.P.G.M., Edenius. L., Ericsson, G., Widemo, F., Wam, H.K. Varied diets, including broadleaved forage, are important for a large herbivore species inhabiting highly modified landscapes. Supplementary Appendix

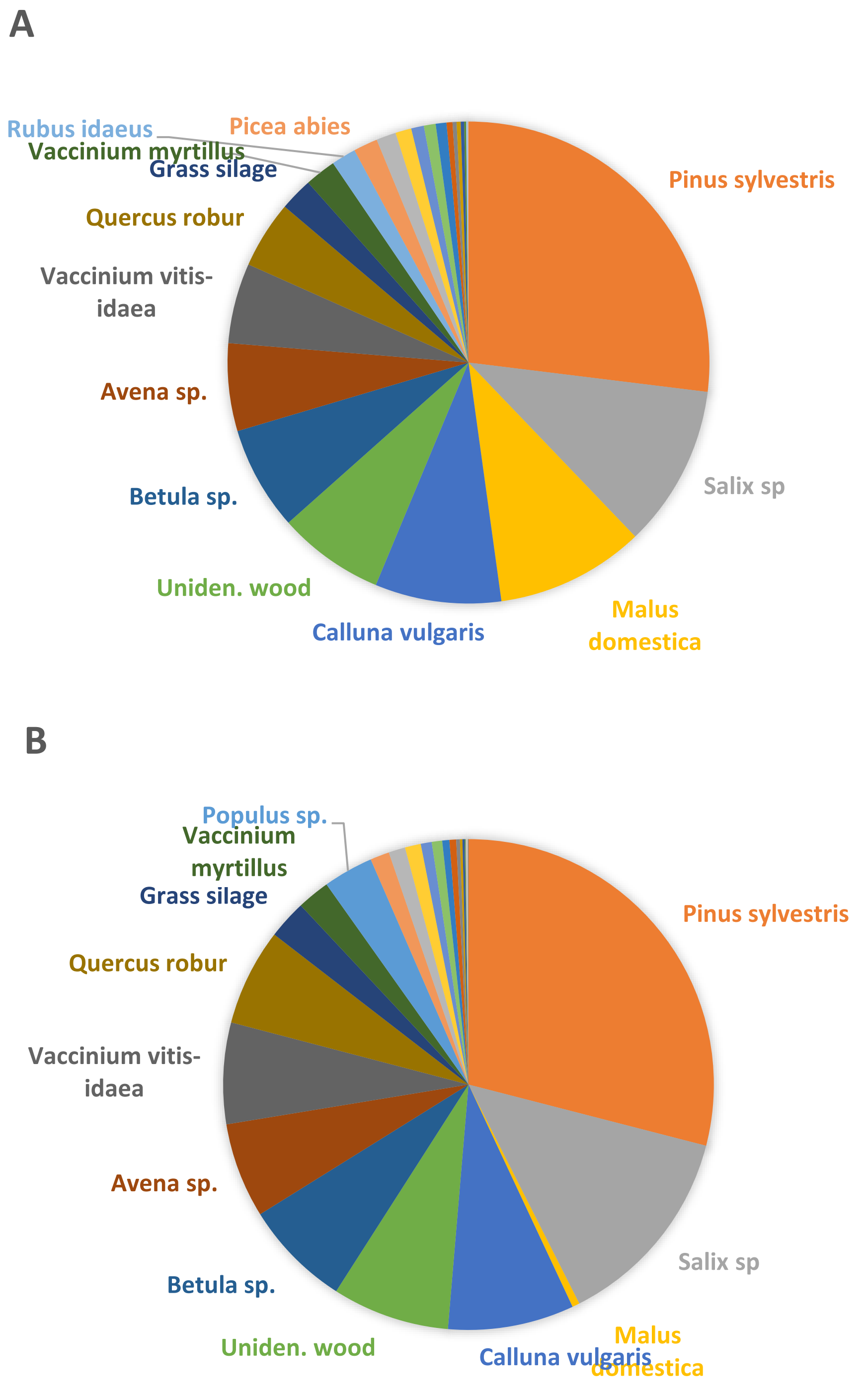

Figure 4SI. Comparison of the mean composition of plant categories, in terms of % dry matter (dm), identified in a first subportion (A) and a second subportion (B) of the same 16 original rumen samples. Plant categories that represent at least 2% of total dm are described by name.

Felton, A. M., Holmström, E., Malmsten, J., Felton, A., Cromsigt, J.P.G.M., Edenius. L., Ericsson, G., Widemo, F., Wam, H.K. Varied diets, including broadleaved forage, are important for a large herbivore species inhabiting highly modified landscapes. Supplementary Appendix

Supplementary Tables

Table 1. Samples from moose in southern Sweden were collected in seven (A-G, see Fig. 1 Main text) moose management areas (MMA, each representing a moose population), divided into multiple moose management units (MMU; subpopulation). The number of MMU from which we obtained samples is indicated ([a]), as well as their summed area ([b]) in terms of tha (1000 ha), mean area ([c]) and the proportion of the MMA which was included in the study ([d]). Samples were collected from moose harvested 23-Oct-2014 and 22-Feb-2015, with the majority during Oct – Nov (some samples lacked date information ([e])). Of the total number of obtained samples ([f]), the proportion representing calves is indicated ([g]). The number of samples ([h]) from these seven moose populations, used for macroscopy analysis of rumen contents, was 302 (21 of the 323 individuals for which we obtained macroscopy results came from areas outside of the seven main study populations).* In the multivariate analysis of diet composition (Fig. 4 Main text) results from 252 rumen samples were used (only including MMUs for which we had at least 5 rumen samples with macroscopy results).

**Moose management area (MMA)**	**A**	**B**	**C**	**D**	**E**	**F**	**G**	**Total**
Area (tha)	73	487	87	141	130	87	169	1176
Number of MMU[a]	6	18	4	8	9	6	9	60
Sum MMU area (tha)[b]	61	245	70	141	108	76	91	793
Mean area of MMU (tha)[c]	10	14	18	18	12	13	10	
Included area (%)[d]	84	50	81	100	83	87	54	
Oct # individuals	6	12	1	21	19	5	28	92
Nov # individuals	15	34	31	43	29	17	16	185
Dec # individuals	12	16	9	14	10	10	6	77
Jan # individuals	13	5	8	7	7	2	5	47
Feb # individuals	1	0	0	1	3	3	1	9
# other individuals[e]	6	3	1	0	6	0	0	16
Total # individuals[f]	53	70	50	86	74	37	56	426
%calves [g]	44	38	80	59	65	47	48	
# macroscopy samples[h]	35	47	36	67	42	29	46	302*

Felton, A. M., Holmström, E., Malmsten, J., Felton, A., Cromsigt, J.P.G.M., Edenius. L., Ericsson, G., Widemo, F., Wam, H.K. Varied diets, including broadleaved forage, are important for a large herbivore species inhabiting highly modified landscapes. Supplementary Appendix

Table 2SI. Mean proportion of dry matter of different plant categories identified by macroscopy of rumen contents for 26 moose subpopulations (MMU) belonging to one of seven populations (MMA A-G; "All" = overall mean). Included are plant categories (23) that represented ≥ 10% dm in ≥ one rumen sample, and occurred ≥ 2.5% of all samples analysed. For each MMU the mean calf carcass body mass (Calf), the number of rumen samples (N) and total number of plant categories identified (N_{pc}) are listed. Plant categories are sorted first according to broad class then alphabetically. Broad classes include broadleaved trees (^{B}), conifer trees (^{C}) and supplementary food (^{S}) and other (no superscript).

MMA	MMU	Calf	N	N_{pc}	^{B}Bet sp	^{B}Pop sp	^{B}Que rob	^{B}Sal sp	^{C}Jun com	^{C}Pic abi	^{C}Pin syl	^{S}Bet vul*	^{S}Gra sil*	^{S}Mal dom*
**All**		58.6	10	18	5.4	1.5	7.1	9.3	1.3	2.1	28.3	0.3	1.7	0.8
**A**	40	54.3	10	20	6.2	0.3	10.1	1.4	6.9	18.0	27.5	0.0	4.5	0.9
**A**	37	53.5	10	12	0.9	0.0	3.8	0.0	1.8	0.0	48.3	0.0	27.0	0.0
**A**	35	51.3	6	9	7.9	0.0	0.6	0.0	7.1	0.0	64.1	0.0	0.0	0.0
**B**	51	54.1	8	21	1.7	2.6	13.7	1.7	0.0	5.0	7.4	0.0	6.4	0.0
**B**	56	64.8	5	18	5.1	0.0	5.6	17.0	1.4	5.8	12.8	0.0	0.0	0.0
**B**	50	59.7	6	17	0.7	0.0	3.4	17.9	0.1	10.1	10.0	0.0	0.0	11.2
**B**	52	59.5	7	20	5.8	4.1	5.8	1.4	1.4	0.2	41.7	0.0	0.0	0.0
**C**	44	56.4	12	18	7.6	0.0	4.4	2.6	4.8	0.6	43.3	0.0	0.0	0.0
**C**	25	52.9	16	20	0.5	0.0	25.2	2.4	4.7	4.2	36.3	1.6	0.0	0.0
**C**	42	52.9	7	18	2.5	0.0	20.0	0.6	1.1	0.0	42.9	0.0	0.0	0.0
**D**	6	63.7	10	14	13.2	0.0	4.1	9.7	0.1	0.0	23.0	0.0	0.0	0.0
**D**	1	62.8	9	17	11.4	2.0	8.2	4.7	0.1	0.0	53.1	0.0	0.0	0.0
**D**	5	61.4	7	18	3.8	2.2	11.8	12.8	0.1	1.4	23.7	0.0	0.0	0.0
**D**	7	59.2	22	27	9.7	0.8	4.9	14.3	0.6	0.0	18.2	0.0	0.0	0.5
**D**	62	58.5	7	13	4.2	0.0	0.8	7.7	0.2	1.4	16.1	0.0	0.0	0.0
**D**	3	59.5	7	16	1.7	0.0	0.8	0.9	1.9	0.1	35.2	0.0	0.0	0.0
**E**	15	62.8	11	21	7.2	0.0	5.0	6.5	0.4	0.0	17.2	0.0	1.5	0.0
**E**	12	62.0	5	17	14.1	2.9	8.8	30.5	0.0	0.0	16.8	0.0	0.0	0.0
**E**	13	60.2	9	15	9.1	6.1	12.9	11.2	0.2	0.0	17.1	0.0	0.0	0.0
**E**	16	60.2	11	23	1.3	1.0	11.3	26.1	0.0	0.2	9.3	0.0	2.0	3.5
**F**	23	68.8	5	13	0.0	4.8	3.3	29.9	0.0	0.0	33.6	0.0	0.0	0.0
**F**	22	59.5	13	20	12.3	1.6	3.8	15.1	0.1	5.6	34.4	0.0	0.7	1.4
**F**	21	59.1	9	13	5.6	6.5	7.0	8.1	0.0	0.0	47.3	0.0	0.0	0.0
**G**	33	57.2	23	27	2.2	0.0	7.9	7.3	0.0	1.6	14.8	1.8	1.3	2.6
**G**	31	56.2	6	22	0.7	0.9	0.5	5.8	0.0	0.6	23.1	2.2	0.0	0.0
**G**	34	53.0	11	20	4.3	2.0	1.6	6.9	0.0	0.9	19.6	3.2	0.0	0.0

Table 2SI continued

MMA	MMU	Bra sp*	Cal vul	Grass 1	Grass 2	Grass 3	Rho tom	Rib sp	Rub ida	Uni bar	Uni pla	Uni woo	Vac myr	Vac vit
**All**		0.5	9.4	0.7	3.5	0.9	0.3	0.2	0.5	0.8	0.3	5.6	7.8	9.1
**A**	40	4.2	4.9	0.1	0.0	0.0	0.0	0.0	0.0	2.4	0.0	6.3	3.8	2.1
**A**	37	0.0	3.3	0.0	0.0	0.1	0.0	0.0	0.0	0.0	0.0	4.5	7.7	2.5
**A**	35	0.0	6.1	0.0	0.0	0.1	0.0	0.0	1.4	0.0	0.0	0.0	8.5	4.3
**B**	51	0.0	8.1	0.0	2.4	0.3	0.0	1.7	3.1	0.2	0.7	7.4	9.1	14.1
**B**	56	5.6	1.3	0.0	0.0	0.7	2.5	0.0	0.0	1.1	0.5	6.6	16.3	14.8
**B**	50	0.0	10.6	0.1	22.7	0.0	0.0	0.0	0.0	0.7	0.0	5.9	1.1	5.2
**B**	52	0.5	6.7	0.4	8.4	0.1	0.0	0.0	0.6	0.0	0.4	6.9	7.8	7.5
**C**	44	0.0	8.5	0.3	0.0	1.2	0.0	0.0	0.2	6.7	0.6	1.7	10.5	6.7
**C**	25	0.3	16.3	0.0	0.0	0.0	0.0	0.0	0.0	0.3	0.0	3.1	3.2	1.5
**C**	42	0.0	12.5	0.1	0.3	0.0	0.0	0.0	0.2	1.8	0.0	1.3	14.6	0.4
**D**	6	0.0	11.9	0.0	7.2	1.0	2.3	0.0	0.6	0.0	0.0	6.3	5.5	15.0
**D**	1	0.0	2.0	0.0	1.9	0.1	0.0	0.1	0.0	0.0	0.1	3.4	4.6	8.1
**D**	5	0.0	15.8	0.2	0.0	3.7	0.0	0.1	1.0	0.0	0.5	5.9	7.3	9.4
**D**	7	0.0	8.4	3.0	4.8	2.4	0.1	0.2	0.1	0.3	0.3	9.4	8.5	13.3
**D**	62	0.0	22.6	0.0	7.3	3.0	0.0	0.0	0.0	0.0	0.0	3.6	14.6	18.1
**D**	3	0.0	15.6	10.5	0.0	4.6	0.0	0.1	0.0	0.0	0.0	1.8	6.3	20.4
**E**	15	0.0	12.8	0.0	2.3	0.4	0.0	0.3	0.3	1.6	0.5	6.9	19.0	17.5
**E**	12	0.0	1.2	0.0	6.8	0.0	0.1	1.9	1.0	0.5	0.3	7.0	2.4	4.6
**E**	13	0.0	11.0	0.1	0.0	0.2	0.6	0.0	0.5	0.5	0.0	15.1	9.8	4.7
**E**	16	0.0	10.4	0.0	1.8	0.9	0.0	0.0	2.5	0.4	1.1	8.9	3.1	15.8
**F**	23	0.0	7.5	0.0	7.3	0.2	0.0	0.4	0.0	0.6	1.2	6.0	0.0	4.8
**F**	22	0.0	3.1	0.7	1.4	0.1	0.0	0.0	2.6	0.0	1.7	4.3	2.4	7.7
**F**	21	0.0	1.7	0.0	2.1	0.5	0.0	0.0	0.0	0.0	0.0	13.0	0.8	7.1
**G**	33	0.0	9.1	0.0	1.9	1.9	0.0	3.4	6.5	0.1	0.6	9.1	5.7	15.6
**G**	31	0.0	23.8	0.0	0.0	1.3	0.0	2.8	0.2	0.3	0.3	7.2	5.4	10.9
**G**	34	0.0	9.2	1.0	0.0	5.6	0.0	1.4	1.7	0.1	2.0	8.7	7.0	23.5

Bet sp. = *Betula* sp.; Pop sp = *Populus* sp.; Que rob = *Quercus robur*; Sal sp = *Salix* sp.; Jun com = *Juniperus communis*; Pic abi = *Picea abies*; Pin syl = *Pinus sylvestris*; Bet vul = *Beta vulgaris*; Gra sil = Grass silage; Mal dom = *Malus domestica*; Bra sp = *Brassica* sp; Cal vul = *Calluna vulgaris*; Grass 1 = grass from forest; Grass 2 = grass from field; Grass 3 = narrow-leaved grass; Rho tom = *Rhododendron tomentosum*; Rib sp = *Ribes* sp.; Rub ida = *Rubus idaeus*; Uni bar = Unidentified bark; Uni pla = Unidentified plant parts; Uni woo = Unidentified wood; Vac myr = *Vaccinium myrtillus*; Vac vit = *Vaccinium vitis-idaea.* * Cultivated crops.

Table 3SI. Principal component coefficients of the mean diet composition of 26 moose subpopulations (moose management units) in southern Sweden, sampled the winter 2014/15. For each subpopulation the mean percentage of dry matter per plant category (15 categories) identified by macroscopy of rumen samples is included, as is the mean calf BM. Values > 0.2 and values < -0.2 on PC1 and PC2 are indicated in bold, as these were interpreted as being those plant categories (also bold) that contributed most strongly to explaining the variation in the clustering of diet types.

Variable	PC1	PC2	PC3
***Populus* spp.**	**0.22**	**0.31**	-0.18
Betula spp.	0.07	0.11	-0.49
Vaccinium myrtillus	-0.11	**-0.33**	-0.07
**Grass from field**	**0.26**	0.11	0.10
Grass silage	-0.20	0.07	0.25
Quercus robur	-0.09	**0.25**	0.27
Juniperus communis	**-0.42**	0.01	-0.05
Picea abies	-0.10	0.13	0.46
Vaccinium vitis idaea	**0.28**	**-0.39**	-0.01
Calluna vulgaris	0.01	**-0.39**	0.23
**Uniden. wood**	**0.34**	0.15	0.18
***Salix* spp.**	**0.41**	0.16	0.04
**narrow-leaved grass**	0.14	**-0.46**	-0.05
Pinus sylvestris	**-0.31**	0.11	-0.48
**Mean calf BM**	**0.38**	0.12	-0.17
**% Variance**	24.2	18.4	11.1

Table 4SI. Hunter-reported body mass (BM) data for moose calves from the Swedish national online data base www.algdata.se, and hunter-reported observational data regarding the observed number of calves per moose cow (Obs calf), and the proportion of females observed with at least one calf (Obs fem), from the database www.viltdata.se, showing 5-year averages for five hunting seasons (2012/13 – 2016/17) per moose study population (Moose Management Area A-G, see Fig. 1 Main text). The comparable data for moose calf BM (II, right column) from this study represent only the hunting season 2014/15.

MMA	I) calf BM (N)	Obs calf (N)	Obs fem (N)	II) calf BM (N)
**A**	56.5 (23)	0.57 (15)	0.46 (15)	51.8 (16)
**B**	59.4 (331)	0.74 (46)	0.54 (46)	65.2 (26)
**C**	53.1 (61)	0.67 (75)	0.51 (75)	52.3 (32)
**D**	60.9 (162)	0.82 (57)	0.61 (57)	59.6 (52)
**E**	60.7 (185)	0.82 (41)	0.60 (41)	58.4 (35)
**F**	62.7 (87)	0.89 (32)	0.62 (32)	60.2 (17)
**G**	54.0 (71)	0.62 (55)	0.50 (55)	54.9 (26)

I) calf BM = mean calf carcass BM as a 5-year average of hunter-reported data to national database (source: www.algdata.se), with the mean number of calves reported per year in brackets; Obs calf = 5-year average observed number of calves per cow moose (source: www.viltdata.se), with the mean number of cow moose observed per year in brackets; Obs fem = 5-year average proportion of females observed with at least one calf (source: www.viltdata.se), with the mean number of cow moose observed per year in brackets; II) calf BM = mean calf carcass BM based on data obtained in this study (2014/15), with the samples size in brackets.

Felton, A. M., Holmström, E., Malmsten, J., Felton, A., Cromsigt, J.P.G.M., Edenius. L., Ericsson, G., Widemo, F., Wam, H.K. Varied diets, including broadleaved forage, are important for a large herbivore species inhabiting highly modified landscapes. Supplementary Appendix

Table 5SI. Multivariate modelling, which relates landscape scale habitat variables to mean body mass of moose calves (BM, dressed carcass kg) and the likelihood of these calves having particularly low BM (N=222 calves, Southern Sweden, Oct 2014-Feb 2015). Individuals were classified as low-weight if the BM was at least 0.5 standard deviation below the mean BM of all calves: <51.9 kg. We ran models with subpopulations nested and not nested with population as a random intercept effect. Forest data was collected from GIS data of a circular area with r=10 km from the centre point of each subpopulation. Models shown here are the most and next most parsimonious according to stepwise selection by AIC, plus the same models with all main effects, but no interactions. We also show full model results for the most parsimonious non-nested model where calf BM is the response variable, because this differed from the nested model. Full model results for the most parsimonious nested model of both response variables are shown in Table 2, main text. F=fast-growing broadleaves (mainly *Betula* spp.). S=slow-growing broadleaves (mainly *Quercus* spp. and *Fagus sylvatica*).

**Model description**						
**Response = calf body mass (kg)**	*Non-nested models* [a]			*Nested models* [b]		
(Intercept)	x	x	x	x	x	x
% forest of total area (pfor)	x		x		x	x
% forest being F broadleaves (fgbl)	x		x		x	x
% of forest being young (yfor)	x	x	x	x	x	x
% forest being S broadleaves (sgbl)	x		x		x	x
pfor*fgbl	x				x	
yfor*sgbl	x				x	
AIC	1737.7	1740.8	1744.6	1738.8	1739.4	1743.9
**Response = low-weight calves**	*Non-nested models* [c]			*Nested models* [d]		
(Intercept)	x	x	x	x	x	x
% forest of total area (pfor)	x	x	x	x	x	x
% forest being F broadleaves (fgbl)			x			x
% of forest being young (yfor)	x	x	x	x	x	x
% forest being S broadleaves (sgbl)						
pfor*fgbl						
yfor*sgbl						
pfor*yfor		x			x	
AIC	246.7	248.4	248.5	248.6	250.1	250.5

[a] mixed model with population (N=7) as random intercept variable. Model selection (comparing models with different fixed, but the same random effects) was done with ML fitting.
[b] mixed model with subpopulation (N=39) nested in population (N=7) as random intercept, otherwise as [a].
[c] logit regression, binomial, with population (N=7) as random intercept
[d] logit regression, binomial with subpopulation (N=39) nested in population (N=7) as random intercept

Felton, A. M., Holmström, E., Malmsten, J., Felton, A., Cromsigt, J.P.G.M., Edenius. L., Ericsson, G., Widemo, F., Wam, H.K. Varied diets, including broadleaved forage, are important for a large herbivore species inhabiting highly modified landscapes. Supplementary Appendix

Result for the most parsimonious non-nested model

**Response = calf body mass (kg)**	β	SE	df	t value	p-value
The most parsimonious non-nested model					
(Intercept)[e]	-2.7	21.1	208	-0.1	0.900
% forest of total area (pfor)	74.0	31.3	208	2.4	0.019
% forest being F broadleaves (fgbl)	235.3	116.9	208	2.0	0.045
% of forest being young (yfor)	190.3	55.2	208	3.4	0.001
% forest being S broadleaves (sgbl)	112.0	54.8	208	2.0	0.042
pfor*fgbl	-542.8	194.9	208	-2.8	0.006
yfor*sgbl	-672.4	302.5	208	-2.2	0.027
The same model in nested version					
(Intercept)[f]	0.4	23.3	183	0.0	0.988
% forest of total area (pfor)	68.5	35.6	25	1.9	0.066
% forest being F broadleaves (fgbl)	224.6	129.3	25	1.7	0.095
% of forest being young (yfor)	184.6	61.4	25	3.0	0.006
% forest being S broadleaves (sgbl)	104.7	63.3	25	1.7	0.111
pfor*fgbl	-510.9	222.1	25	-2.3	0.030
yfor*sgbl	-645.9	344.6	25	-1.9	0.073

[e] mixed model with population (N=7) as random intercept variable. Model selection (comparing models with different fixed, but the same random effects) was done with ML fitting, but the final model is presented with REML as this is considered to give more precise estimates of the coefficients.
[f] mixed model with subpopulation (N=39) nested in population (N=7) as random intercept, otherwise as [e]

Felton, A. M., Holmström, E., Malmsten, J., Felton, A., Cromsigt, J.P.G.M., Edenius. L., Ericsson, G., Widemo, F., Wam, H.K. Varied diets, including broadleaved forage, are important for a large herbivore species inhabiting highly modified landscapes. Supplementary Appendix

Table 6SI. Principal component coefficients of the composition of plant categories in 302 individual moose rumen samples, sampled in southern Sweden the winter 2014/15. For each individual the percentage of dry matter per plant category (15 categories) identified by macroscopy is included, and the code of their respective age-sex class (calf = 1, yearling = 2, adult female = 3, adult male = 4).

**Variable**	**PC1**	**PC2**	**PC3**
Betula spp.	-0.045	-0.066	-0.016
Broad-leaved grass from field	0.08	-0.06	0.213
Calluna vulgaris	-0.223	0.151	-0.183
Grass silage	0.011	-0.119	0.31
Juniperus communis	-0.04	0.07	0.098
narrow-leaved grass	0	0.372	-0.152
Picea abies	0.083	-0.098	0.489
Pinus sylvestris	-0.24	-0.409	-0.315
Populus spp.	0.252	0.045	-0.25
Quercus robur	0.283	-0.045	0.039
Root vegetables	0.055	-0.019	0.132
Salix spp.	0.219	0.053	-0.114
Uniden. wood	0.379	0.115	-0.171
Vaccinium myrtillus	-0.157	0.341	0.161
Vaccinium vitis-idaea	-0.13	0.384	0.104
Age-sex class (1-4)	0.005	0.045	-0.183
**% Variance**	**12.4**	**9.6**	**8.4**

References cited in Supplementary Information

CadasterENVSweden (2015) CadasterENV Sweden - a multicale and multipurpose land cover monitoring system. Swedish Environmental Protection Agency Stockholm.

Cederlund, G., Ljungqvist, H., Markgren, G. & Stålfelt, G. (1980) Foods of moose and roe-deer at Grimsö in central Sweden: results of rumen content analyses. *Swedish Wildlife Research,* **11,** 171-247.

Morellet, N., Klein, F., Solberg, E.J. & Andersen, R. (2011) The census and management of populations of ungulates in Europe. *Ungulate management in Europe: problems and practices* (eds R.J. Putman, M. Apollonio & R. Andersen), pp. 106–143. Cambridge University Press, Cambridge, UK.

Mossberg, B. & Stenberg, L. (2003) *Den nya nordiska floran*. Walström & Widstrand.

Nichols, R.V., Akesson, M. & Kjellander, P. (2016) Diet Assessment Based on Rumen Contents: A Comparison between DNA Metabarcoding and Macroscopy. *Plos One,* **11**.

RCoreTeam (2017) R: A language and environment for statistical computing. (ed. R.F.f.S. Computing). Vienna, Austria.

Zuur, A.F., Ieno, E.N. & Elphick, C.S. (2010) A protocol for data exploration to avoid common statistical problems. *Methods in Ecology and Evolution,* **1,** 3-14.
